# Ancestral Sequence Reconstruction as a Tool to Detect and Study De Novo Gene Emergence

**DOI:** 10.1093/gbe/evae151

**Published:** 2024-07-15

**Authors:** Nikolaos Vakirlis, Omer Acar, Vijay Cherupally, Anne-Ruxandra Carvunis

**Affiliations:** Institute for Fundamental Biomedical Research, BSRC “Alexander Fleming”, Vari, Greece; Pittsburgh Center for Evolutionary Biology and Medicine, Department of Computational and Systems Biology, School of Medicine, University of Pittsburgh, Pittsburgh, PA 15213, USA; Pittsburgh Center for Evolutionary Biology and Medicine, Department of Computational and Systems Biology, School of Medicine, University of Pittsburgh, Pittsburgh, PA 15213, USA; Pittsburgh Center for Evolutionary Biology and Medicine, Department of Computational and Systems Biology, School of Medicine, University of Pittsburgh, Pittsburgh, PA 15213, USA

**Keywords:** de novo gene emergence, ancestral sequence reconstruction, budding yeast, novel genes, phylostratigraphy

## Abstract

New protein-coding genes can evolve from previously noncoding genomic regions through a process known as de novo gene emergence. Evidence suggests that this process has likely occurred throughout evolution and across the tree of life. Yet, confidently identifying de novo emerged genes remains challenging. Ancestral sequence reconstruction is a promising approach for inferring whether a gene has emerged de novo or not, as it allows us to inspect whether a given genomic locus ancestrally harbored protein-coding capacity. However, the use of ancestral sequence reconstruction in the context of de novo emergence is still in its infancy and its capabilities, limitations, and overall potential are largely unknown. Notably, it is difficult to formally evaluate the protein-coding capacity of ancestral sequences, particularly when new gene candidates are short. How well-suited is ancestral sequence reconstruction as a tool for the detection and study of de novo genes? Here, we address this question by designing an ancestral sequence reconstruction workflow incorporating different tools and sets of parameters and by introducing a formal criterion that allows to estimate, within a desired level of confidence, when protein-coding capacity originated at a particular locus. Applying this workflow on ∼2,600 short, annotated budding yeast genes (<1,000 nucleotides), we found that ancestral sequence reconstruction robustly predicts an ancient origin for the most widely conserved genes, which constitute “easy” cases. For less robust cases, we calculated a randomization-based empirical *P*-value estimating whether the observed conservation between the extant and ancestral reading frame could be attributed to chance. This formal criterion allowed us to pinpoint a branch of origin for most of the less robust cases, identifying 49 genes that can unequivocally be considered de novo originated since the split of the *Saccharomyces* genus, including 37 *Saccharomyces cerevisiae*-specific genes. We find that for the remaining equivocal cases we cannot rule out different evolutionary scenarios including rapid evolution, multiple gene losses, or a recent de novo origin. Overall, our findings suggest that ancestral sequence reconstruction is a valuable tool to study de novo gene emergence but should be applied with caution and awareness of its limitations.

**Significance**

During evolution, entirely novel genes can arise “from scratch” in regions of the genome that previously had no function as genes. Accurately detecting such genes and studying the process by which they evolved are challenging. One approach to do so is to computationally reconstruct the ancestral state of a novel gene, but the robustness and suitability of this approach are unclear. Here, we constructed a computational workflow to apply this ancestral sequence reconstruction approach to short genes in budding yeast. We found that it performs well in capturing the ancient origins of widely conserved genes while also providing solid evidence for the recent evolutionary origin of a small number of cases. However, this approach should be applied with caution and awareness of its limitations, since it gives ambiguous results for approximately a quarter of cases.

## Introduction

How new genes originate is a fundamental question in biology because genetic novelty underlies molecular, phenotypic, and organismal novelty (Kaessmann 2010). Understanding how and when novel genes arise is therefore essential to understand evolution at every level of biological organization. For a long time, new genes and protein functions were believed to result exclusively through tinkering and recombination, using preexisting genes and gene parts as raw material (Tautz 2014). Consequently, processes such as substitutions, duplication, and divergence, gene fusion and fission, exon shuffling, or horizontal gene transfer (HGT) have been extensively studied and their importance is established.

Nonetheless, a radically different route to genetic novelty exists: a novel gene can evolve from entirely noncoding sequences in a process known as de novo gene emergence (McLysaght and Guerzoni 2015; Oss and Carvunis 2019). Long considered so improbable as to be impossible (Jacob 1977), de novo gene emergence has high potential to produce an entirely new protein function since noncoding sequences are free of the constraints acting on preexisting coding sequences. De novo genes have been found in every eukaryotic lineage studied so far and can have central cellular functions (McLysaght and Guerzoni 2015). This has led to de novo emergence being increasingly viewed as a universal evolutionary mechanism.

It is challenging to distinguish whether a new gene has emerged de novo or through other evolutionary processes. Indeed, rapid sequence divergence beyond recognition following events such as duplications or rearrangements, as well as HGT, can also result in a gene appearing to be novel or taxonomically restricted (Vakirlis, Carvunis, et al. 2020; Weisman et al. 2020). It is thus important to develop robust methods for distinguishing between these different evolutionary routes, in order to assess the impact of de novo gene emergence and study the characteristics and function of de novo genes.

Evidence for de novo gene emergence can be provided by computational comparative genetics approaches (Vakirlis and McLysaght 2019). The strongest kind of evidence is confidently inferring that the genomic locus that now harbors a novel gene in a given lineage did not encode a protein sequence in the past. Until recently, the gold standard was to align the novel gene to its orthologous regions in multiple out-group species and demonstrate that these out-group loci were noncoding by identifying specific mutations that enabled the presence of an open reading frame (ORF) in the genome of interest. Parsimoniously one can then infer that the ancestral state of the positions in question was such that disrupted the ORF currently present in the focal lineage.

However, visually inspecting such alignments does not always result in clear-cut conclusions. It is especially delicate to draw robust conclusions when the candidate new genes are short and when alignments contain frameshifts. In such tricky cases, conclusions are typically drawn based on a personal judgement call rather than a formal test. A potentially more powerful and accurate approach is to use ancestral sequence reconstruction (ASR). ASR allows both estimating whether an ancestral sequence contained an ORF or not, as well as tracing the mutational transition from noncoding to coding. Thus, the application of ASR to the task of de novo gene detection could be a potent tool to gain insights into this evolutionary process.

ASR has mostly been used in the context of protein alignments in order to reconstruct ancestral protein sequences from extant ones (Hochberg and Thornton 2017). Only a handful of studies have applied ASR to de novo genes (Vakirlis, Acar, et al. 2020; Lange et al. 2021; Papadopoulos et al. 2021; Vakirlis et al. 2018, 2022; Peng and Zhao 2024; Sandmann et al. 2023) thus far, and an in depth assessment of its performance and limitations is lacking. Here, we evaluate how reliably existing ASR tools can estimate the emergence date of short ORFs annotated in the budding yeast *Saccharomyces cerevisiae*'s genome. Using reading frame conservation (RFC) between an ORF and its inferred ancestor as a quantitative measure of ORF age, we conclude that ASR allows robust evolutionary inference for ancient genes but should be used with caution to infer recent events of de novo gene emergence.

## Materials and Methods

### Description of the Data Set

Our initial data set consisted of 2,816 annotated protein-coding genes of *S. cerevisiae* that were included in the data set analyzed by Vakirlis, Acar, et al. (2020) and were under 1,000 nt long. The orthologous regions for each of these genes in seven *Saccharomyces* species (*Saccharomyces paradoxus*, *Saccharomyces mikatae*, *Saccharomyces kudriavzevii*, *Saccharomyces uvarum*, *Saccharomyces jurei*, *Saccharomyces arboricola*, and *Saccharomyces eubayanus*) were identified as follows: first, genomes were obtained from the following sources: *S. paradoxus* from Liti et al. (2009), *S. arboricolus* from Liti et al. (2013), *S. jurei* from Naseeb et al. (2017), and *S. mikatae*, *S. bayanus var. uvarum*, *S. eubayanus*, and *S. kudriavzevii* from Scannell et al. (2011). Alignments were constructed between each *S. cerevisiae* ORF and its homologs in each *Saccharomyces* relative using synteny information. To identify anchor genes for syntenic blocks, BLASTP (Altschul et al. 1997) was run for each annotated ORF in *S. cerevisiae* against each ORF in the comparison species. Identified homolog pairs with *E* < 10^−7^ were selected as potential anchors. For each ORF in the *S. cerevisiae* genome, the upstream anchor G0 and downstream anchor G1 were selected that minimized the sum of the distance between the anchors in *S. cerevisiae* and the distance between the anchors in the comparison species; this sum was required to be less than 60 kb. The sequence between and including G0 and G1 was then extracted from both the *S. cerevisiae* genome and the comparison species and a pairwise alignment of the syntenic region was generated using MUSCLE v.3.8.31. Multiple sequence alignments of the exact *S. cerevisiae* gene locus to its orthologous *Saccharomyces* genomic regions were generated with MAFFT (Katoh and Standley 2013) using default parameters. We removed 183 *S. cerevisiae* genes from the data set because the orthologous region of *S. cerevisiae* could only be identified in less than six other *Saccharomyces* species, or it contained >1,000 gaps/sequence. For the remaining 2,633 genes, we also generated an alternative, extended version of the multiple sequence alignments by including 500 nt flanking the *S. cerevisiae* ORF downstream and upstream. For each *S. cerevisiae* gene, we collected gene and protein properties from Carvunis et al. (2012) and Vakirlis, Acar, et al. (2020). We also performed protein-level sequence similarity searches for every gene, against a protein sequence database containing all fungal proteomes downloaded from NCBI's RefSeq in May 2021 plus the 332 *Saccharomycotina* proteomes from Shen et al. (2018). This search was conducted with BLASTP (Altschul et al. 1997) using an *E*-value cutoff of 0.001 and the *-max_target_seqs* flag set to 1,000. The results of the similarity searches were processed as in Stavropoulou et al. (2022): for each gene, we first obtained the list of all fungal species with a significant similarity match. Phylogenetic age of each gene was then calculated as the most recent common ancestor of all species with a match. The NCBI Taxonomy common tree was used for this, resulting in classification into the following phylogenetic ages: species-specific, genus (*Saccharomyces*), family (*Saccharomycetaceae*), order (*Saccharomycetales*), division (*Ascomycota*), or kingdom (*Fungi*). For each gene, we also counted the number of species with match (number of species with homologs).

### Phylogenetic Reconstruction

**Fig. 1.**
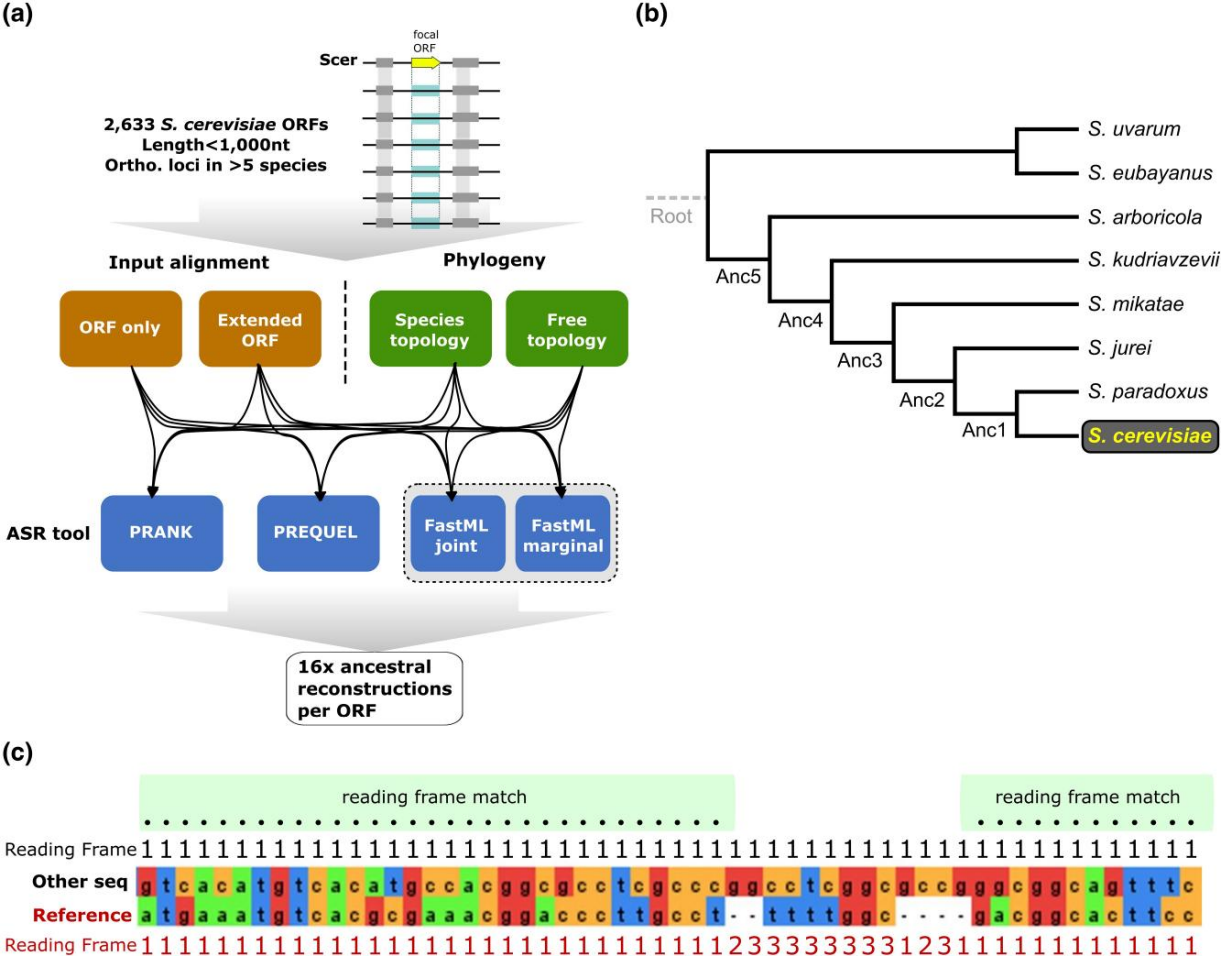
a) Overview of the workflow followed in the present study to generate a set of ASR methodological variations. b) Tree topology of the *Saccharomyces* genus used in our analyses. Reconstructions of the root node (in gray) are not taken into account due to uncertainty of its placement. c) An example alignment to illustrate how the RFC measure is calculated. The reading frame of each sequence and how it changes with the presence of gaps is shown. Numbers (1, 2, and 3) correspond to the three forward reading frames. RFC is calculated by counting nongap positions where the two frames match (marked with blacked dots) and dividing by the length of the reference sequence, which in this study is always the *S. cerevisiae* ORF. In this example, the RFC score would be 43 (number of black dots) divided by the length of the reference sequence, which is 50: 43/50 = 0.86.

Phylogenetic trees were reconstructed using RAxML next generation (Kozlov et al. 2019) (*raxml-ng*) with the GTR substitution matrix, empirically estimated rates and nucleotide frequencies, four categories of rates drawn from a GAMMA distribution with ML inference of its shape parameter using the following command: *raxml-ng --seed 12546582 --model GTR + F + G*. For the species-topology phylogeny we additionally used the species topology as shown in Fig. 1b in newick format with the additional arguments: *--evaluate –tree SACCH_TOP.nwk*. The resulting phylogenetic tree, in the case of the species-topology, was rerooted using *S. eubayanus* and *S. uvarum* (Seub-Suva) as out-groups with the GoTree utility (Lemoine and Gascuel 2021) to ensure downstream consistency. The free topology tree was rerooted at midpoint using GoTree. For use with the ASR tool PREQUEL (see next subsection), a phylogenetic model had to be generated using the PHAST (Hubisz et al. 2011) utility PhyloFit. The trees generated by RAxML were provided to PhyloFit together with the initial input MSA. We then confirmed that the tree and model fitted with PhyloFit were identical to that of RAxML.

### Ancestral Sequence Reconstruction

ASR was performed with FastML (Ashkenazy et al. 2012) using the following command: *perl FastML_Wrapper.pl --MSA_File “INPUT_ALIGNMENT.fasta” --seqType nuc --Tree “INPUT_TREE.nwk” --SubMatrix GTR --OptimizeBL no --indelReconstruction ML --outDir “OUTPUT_DIR”*.

ASR was performed with PRANK (Löytynoja and Goldman 2010) without iterations using the following command:

*prank -d “INPUT_ALIGNMENT.fasta” -support -showall -keep -F -once -o=“OUTPUT_PREFIX” -t=“INPUT_TREE.nwk”*.

ASR was performed with PREQUEL from the PHAST (Hubisz et al. 2011) package using the following command: *prequel “INPUT_ALIGNMENT.fasta” “INPUT_MODEL.phylofit_corTree.mod” OUTPUT_PREFIX*, and then once again with the *-n* argument to obtain the posterior probabilities.

### Identification of ORFs in Ancestral Sequences

For every ancestral sequence in each variation of ASR, we performed the following: first, we identified all ORFs on the forward strand using *getorf* from EMBOSS (Rice et al. 2000) defined either as ATG-STOP or STOP-STOP. The coordinates of each ORF on the ancestral sequence were stored. Then, a pairwise alignment of the entire ancestral sequence and the *S. cerevisiae* extant ORF was generated using the command “*pairwise2.align.globalds (S.cer_sequence, Ancestral_sequence._data, subs_mat, -3, -.1, one_alignment_only = True)*” from Biopython and the coordinates of each ORF were transposed to correspond to the coordinates in the pairwise alignment. Subsequently, the RFC (Kellis et al. 2003; Wacholder et al. 2023) score was calculated for each ancestral ORF based on the pairwise alignment defined as follows: (length covered by the ancestral ORF aligned in the *S. cerevisiae* ORF frame)/(length of the *S. cerevisiae* ORF). For each ancestor of each ASR variation, we kept the ORF with the maximum RFC score.

To select the phylogenetic branch on which an ORF first appeared, based on a predefined RFC cutoff, we performed the following: starting from the root of the phylogenetic tree and moving towards the leaves, we selected the first branch where an ancestral ORF existed with a maximum RFC higher than the predefined cutoff (e.g. 0.6) was selected as the evolutionary origin of the ORF. To select the phylogenetic branch on which an ORF first appeared, based on an empirical *P*-value, we performed the following: first, we computed an empirical *P*-value at each ancestral sequence of each ASR variation, by pseudorandomizing the ancestral sequence 1,000 times using the *random* package in Python and then for each of the 1,000 randomizations, ORFs (using the Stop-Stop definition only) were extracted, RFC was calculated for each one, and the maximum RFC was kept, as described above. This resulted in a set of “randomized” 1,000 best RFC values, representing an empirical null model. Based on this distribution, we then calculated a *P*-value for the real maximum RFC score (one for each ancestral sequence of each ASR variation) by counting the number of randomized values greater than the real one and dividing by 1,000.

### Statistical Analyses

All statistics were done in R v.3.6.2 (R Core Team 2023). Plots were generated using *ggplot2* (Wickham 2011). All statistical details including the type of statistical test performed and exact value of *n* (*n* represents either number of ORFs, sequence reconstructions or ancestors) can be found in the Results and figure legends. Boxplots show median (horizontal line inside the box), first and third quartiles of data (lower and upper hinges), and values no further or lower than 1.5 ∗ distance between the first and third quartiles (upper and lower whisker).

## Results

### A Computational Pipeline to Reconstruct and Conservatively Estimate the Coding Capacity of Ancestral Nucleotide Sequences

We assembled nucleotide multiple sequence alignments of all annotated *S. cerevisiae* ORFs shorter than 1,000 nt (*n* = 2,633; Fig. 1a) with their respective orthologous genomic loci in at least six closely related *Saccharomyces* species (see Materials and Methods and Fig. 1b). These alignments served as inputs for ASR, using a pipeline we designed to test how robust ASR inferences would be to methodological choices. This pipeline combines three different phylogenetic tools, phylogenetic trees built with and without the species topology as a constraint, and two different types of input alignments: one based only on the exact region of the *S. cerevisiae* ORF and one based on an extended region (see Materials and Methods). The phylogenetic tools used are the following: FastML (Ashkenazy et al. 2012), which performs both marginal and joint maximum likelihood (ML) reconstructions of characters and ML reconstruction of indels (hereafter FastML_joint and FastML_marginal); PREQUEL from the PHAST (Hubisz et al. 2011) package, which performs ML reconstruction of characters and parsimonious reconstruction of indels and prefers to infer deletions when insertions and deletions cannot be distinguished (producing an upward bias on the length of the sequence at root); and PRANK (Löytynoja and Goldman 2010), which uses ML reconstruction of characters and a custom algorithm for the inference of insertions and deletions (Fig. 1a).

After running our ASR pipeline on all input alignments, we searched each ancestral sequence for the presence of ORFs that could correspond to ancestral versions of the extant *S. cerevisiae* gene. We excluded the *Saccharomyces* root node from all downstream analyses due to uncertainty about the position of the root node along the root branch (Fig. 1b). The position of the root node can significantly influence the resulting reconstruction: if it sits closer to the out-group, the reconstructed sequence will be more similar to the out-group sequences, whereas if it sits closer to the in-group, the reconstructed sequence will be more similar to in-group sequences. The root reconstruction therefore cannot be trusted in the absence of additional out-groups.

We defined putative ancestral ORFs in two ways: ATG-STOP hereafter “ATG” or STOP-STOP (that is, between two stop codons) hereafter “noATG.” The similarity of each ancestral ORF longer than 30 nt relative to the extant one of *S. cerevisiae* was scored using the RFC measure (Kellis et al. 2003; Wacholder et al. 2023): (length covered by the ancestral ORF aligned in the *S. cerevisiae* ORF frame)/(length of the *S. cerevisiae* ORF). An RFC value = 1 means that an ancestral ORF exists that is at least as long as the *S. cerevisiae* ORF and aligns to it in the same frame and without frameshifts. Figure 1c shows an example alignment with an RFC of 0.86. We inferred whether a *S. cerevisiae* ORF originated de novo since the *Saccharomyces* common ancestor according to each combination of methodological choices implemented in our pipeline, for a range of RFC cutoffs (0.5, 0.6, 0.7, and 0.9).

**Fig. 2.**
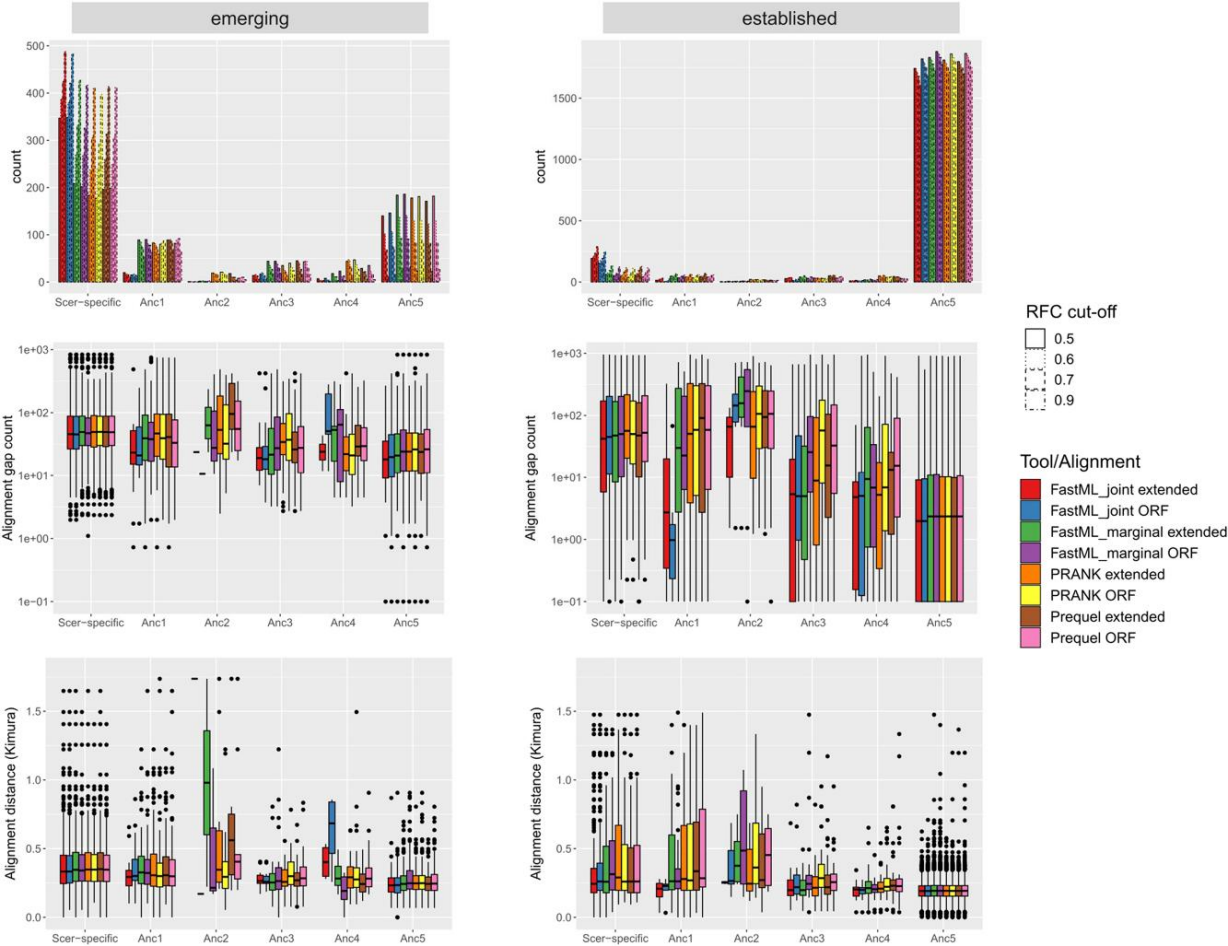
Distributions of branches of origin and input multiple sequence alignment statistics. Top: Distributions of branch where the most ancient ancestral ORF has been identified using four different RFC cutoffs and eight different ASR methodologies (tools + alignment type). Bars within each node (e.g. “Anc5”) correspond, from left to right, to RFC cutoff of 0.5, 0.6, 0.7, and 0.9. Results using species topology and “noATG” definition of ORFs are shown. Results for free topology and ATG definition can be found in supplementary fig. S1, Supplementary Material online. Middle: Distributions of average gap count per sequence in the initial multiple sequence alignment, over the different predicted branches of origin (most ancient ancestral ORF, using an RFC cutoff of 0.6). Bottom: Same as above, but for the average pairwise Kimura distance in the input alignment.

We compared the outputs of ASR with a classification of the input ORFs into emerging or established previously developed by Vakirlis, Acar, et al. (2020) using a combination of sequence and selection signatures. Figure 2 shows the results using the species topology and the definition of ORF without the need for an ATG start codon (“noATG”). Results with free tree topology and using the alternative ORF definition (“ATG”) can be found in supplementary fig. S1, Supplementary Material online.

In agreement with the initial analysis, the vast majority of established ORFs were classified by ASR as ancient and the majority of emerging ORFs were classified by ASR as *S. cerevisiae* specific (Fig. 2, top). The predicted origin of emerging ORFs is more sensitive to the choice of cutoff than that of established ORFs (as evidenced by the height difference of bars of the same color within each ancestral node in Fig. 2, top). The type of initial alignment used as input (ORF only or extended ORF region) had minimal impact, with the vast majority of ORFs being predicted to have the same origin. Note that under a free topology, due to a resulting tree topology that differs from the species one and where Anc5 does not exist, the most ancient ancestor for a number of ORFs predictably shifts to the next available ancestors 3 and 4 (supplementary fig. S1a, Supplementary Material online). When setting an arbitrary RFC cutoff of 0.6, ORFs predicted to be ancient by ASR have initial alignments with shorter genetic distance and more gaps than those predicted to be *S. cerevisiae* specific (Fig. 2, middle and bottom). This is consistent with young ORFs evolving faster, which has been reported before (Albà and Castresana 2005; Cai and Petrov 2010; Moyers and Zhang 2015), but it can also be explained with faster evolving ancient genes whose more “difficult” initial alignments are challenging for ASR.

Overall, ASR methodologies based on the species phylogeny unanimously inferred 1,554 ORFs to be at least as ancient as the earliest ancestor we considered (Anc5), with RFC > 0.9. All but five of these ORFs were also classified as “established” by Vakirlis et al., and, in 1,515/1,554 (97.5%) cases, all downstream ancestors (Anc4, 3, etc.) have an RFC > 0.9. Therefore, the presence of the intact ORF is highly robust throughout the tree and these 1,554 cases can safely be considered ancient. We conclude that ASR performs well in such cases (supplementary table S1, Supplementary Material online).

### An Empirical *P*-Value Allows to Confidently Select a Most Likely Branch of Origin for Many ORFs

**Fig. 3.**
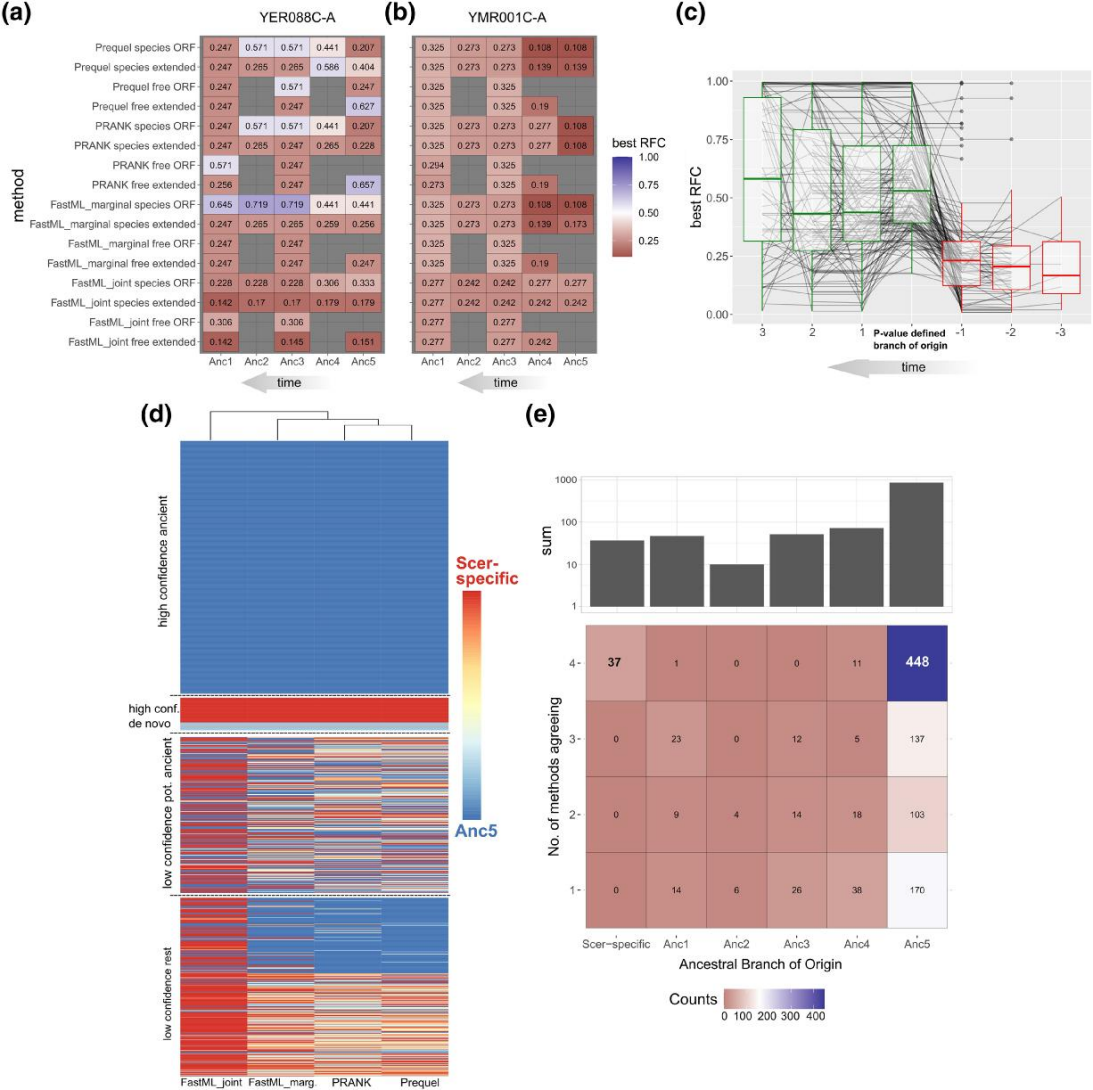
a) One example of maximum RFC score for different ancestors, as predicted using ancestral sequences from different methodologies (tool + phylogeny + inp. alignment). noATG ORF definition shown. Example is gene YER088C-A, a representative case where picking a branch of origin is not immediately obvious since when using an arbitrary RFC cutoff (e.g. 0.6) some methodologies lead to presence of ORFs in much older nodes than others; for example, FastML_marginal + species + ORF would place the origin at Anc3, whereas PRANK + species + extORF would lead to a species-specific origin. b) Another example (YMR001C-A) where no methodology predicts presence of an ancestral ORF, even with an RFC cutoff as low as 0.5, and should thus be considered species-specific. c) Best RFC scores at ancestors before (−1, −2, and −3) and after (1, 2, and 3) the branch of origin, when it is defined based on our empirical *P*-value. Values corresponding to the same gene (149 genes in total) are connected by lines. d) Heatmap showing the ancestral branches of origin as defined using our empirical *P*-value cutoff of 0.01, for each of the 1,076 ORFs that were not previously predicted to be robustly ancient, using four different ASR tools (species topology, ORF-only alignments, noATG ORF definition). Based on the agreement among methodologies, we can group them into high-confidence ancient (top group), high-confidence de novo including *S. cerevisiae*-specific (second from the top), low-confidence but potentially ancient since at least one methodology predicts and ancient origin (third from the top), and the rest (bottom). e) Number of methodologies agreeing in the ancestral branch of origin of each ORF. Same data as in d).

For the remaining 1,076 ORFs, different ASR methodological variations gave at least partly conflicting estimates (YCR039C, YJL077W-B, and YOR202W were removed from the analysis due to missing sequences in at least one of the species, which led to failure of some ASR tools). Two examples of such ORFs and the best RFC scores in ancestral sequences predicted by different methodologies can be found in Fig. 3a. On the left, we show a case where, using an RFC cutoff of 0.5, some methodologies would predict a much more ancient origin than others. On the right, we show a case where no methodology retrieves an ORF with RFC > 0.4 in any ancestor; thus, using an RFC cutoff of 0.5 would result in a coherent classification as species-specific, across ASR methodological variations, but an RFC cutoff of 0.3 would not. These examples illustrate that relying on an arbitrary RFC cutoff for inferring ORF origination is problematic.

We thus asked whether one can systematically infer when an ORF has formed, taking into account the fact that ORFs can form randomly. To this end, we randomized each entire ancestral sequence while keeping its original nucleotide composition and then logged the best RFC-scoring ORF, repeating this procedure 1,000 times (see Materials and Methods). This produced an empirical distribution of values which we then used to assign a *P*-value on the best RFC-scoring ORF of the real ancestral sequence. The most ancient phylogenetic node where a *P*-value of <0.01 was recorded was then kept as the most likely branch of origination of this ORF, that is, the most ancient branch where the presence of such an ORF is unlikely to be due to chance. It is important to stress that, ultimately, this approach may lead to strongly conservative estimates because de novo emergence could start from genomic loci, which do happen to harbor unusually long ORFs, something that is bound to occur given that the genomic space is continuously explored throughout evolutionary time. The relationship between the empirical *P*-value and the best RFC ORFs at the Anc5 ancestor, which appears to have a sigmoid-like shape, can be found in supplementary fig. S2, Supplementary Material online.

If our *P*-value-based branch of origin estimates are evolutionarily meaningful, we expect that *P*-values in ancestors below the one where we predict an ORF originated would also be significant, consistent with an enduring ORF presence. Indeed, this is what we find: we analyzed 967 cases with a predicted branch of origin at Anc5, 4, 3, or 2 (so that at least one ancestor lower can be examined) and found that for 749/967 (77%), all downstream ancestors have a *P*-value of <0.01, and for a further 7.5% all but one downstream ancestors do. There are only 15% of cases with high uncertainty. Thus, relying on the *P*-value to define a branch of origin leads to consistent results downstream of the branch of origin.

An additional prediction is that best RFC scores should be significantly lower in ancestors prior to the branch of origin, compared with those later, and this is what we found. We analyzed 149 cases with a *P*-value-defined branch of origin at Anc4, 3, or 2, allowing for at least one ancestor higher and one ancestor lower of the origin to be examined. We then compared the distributions of best RFC scores in ancestors before (*n* = 202) and after (*n* = 498) the branch of origin (the branch of origin itself included in the latter), and we observed a strong difference in means (0.25 before vs. 0.54 after; Wilcoxon test *P* < 10^−16^). These findings are visualized in Fig. 3c.

An overview of the predictions of branches of origin of the different methodologies using the species topology, when relying on the empirical *P*-value, can be found in Fig. 3d and the raw data can be found in supplementary table S2, Supplementary Material online. Note that since results between the ORF-only and extended-ORF alignments were highly similar, we only use the ORF-only alignments for this and all downstream analyses. When comparing the predictions of the different methodologies, FastML_joint stands out as resulting in more species-specific estimates than the rest (424 ORFs or 39.3%, compared with 12.7% on average for the rest). This is because this method has a strong tendency to infer deletions when encountering gaps in the input alignment, resulting in longer ancestral sequences. No other significant bias among methodologies was found.

We observed that for 442 ORFs, an ancient origin was predicted by all ASR methodologies, and we classified these as “high-confidence ancient” (Fig. 3d, top group). For another 49 ORFs, all methodologies agreed on an origin after the split of the genus and so we can safely conclude that these have emerged de novo (Fig. 3d; “high-confidence de novo”). Notably, 37 of them are *S. cerevisiae* specific. Integrating ASR with a systematic RFC *P*-value, therefore, improved the robustness of evolutionary inferences relative to an arbitrary RFC cutoff. However, the remaining 623 ORFs should be considered “low-confidence” with discordant predictions that suggest uncertainty about when they originated, including 273 potentially ancient ones where only one or two methodologies predict an origin at Anc5. In Fig. 3e, we provide a condensed view of these findings, allowing to compare the numbers of confident predictions as well as those of more uncertain ones. We next asked what could explain this uncertainty.

### Multiple Evolutionary Scenarios Could Account for the Uncertain Origin of Low-Confidence ORFs

**Fig. 4.**
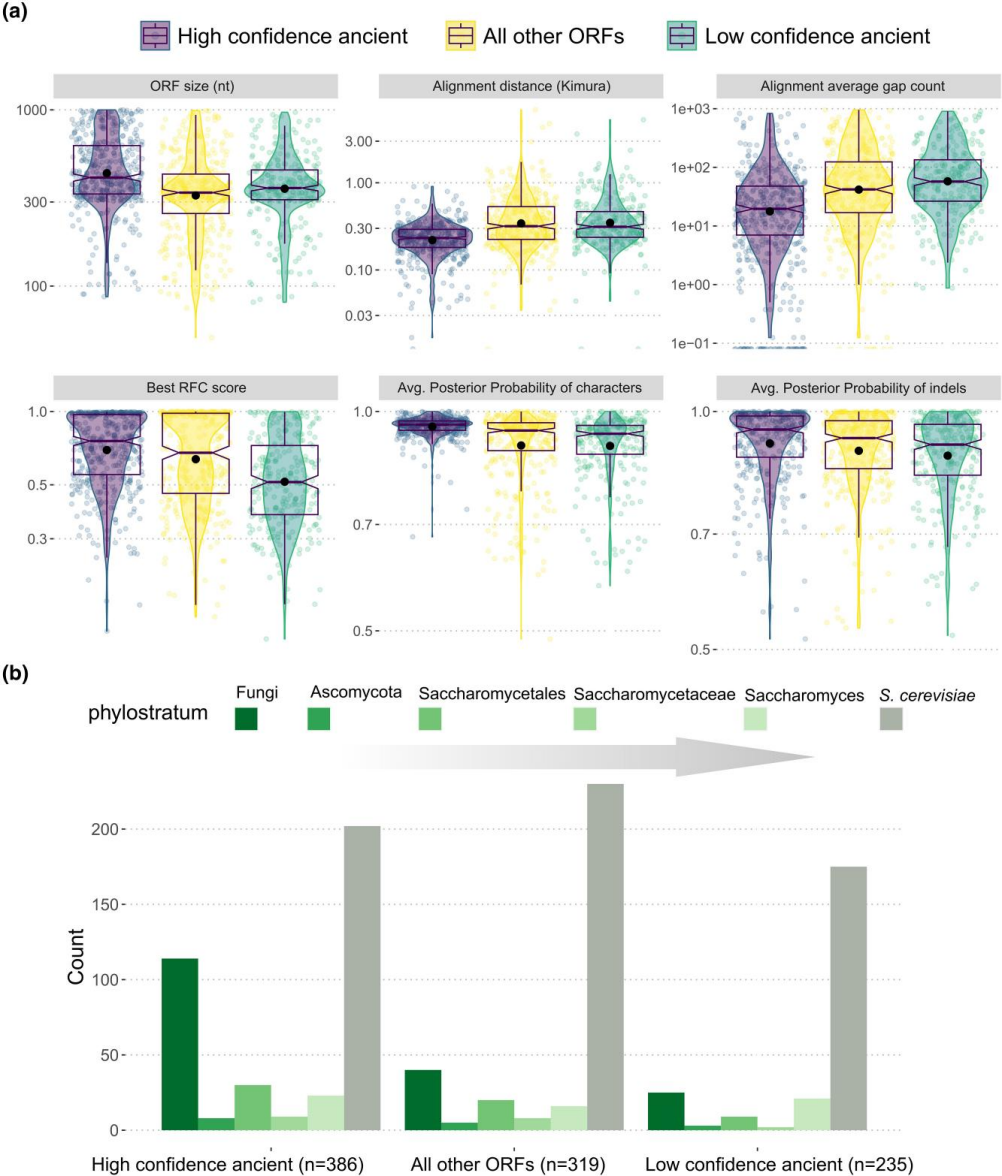
Comparison of low-confidence and high-confidence ancient ORFs and all other remaining ORFs. a) ORF size, best RFC score at Anc5 ancestor, number of gaps per sequence in the initial ORF-only MSA, average pairwise Kimura distance in the initial ORF-only MSA, average posterior probability of characters at the Anc5 ancestor as calculated by FastML_marginal, and average posterior probability of indels as calculated by FastML_marginal. b) Counts of ORFs per major phylostratigraphic origin for high-confidence ancient ORFs, low-confidence ancient ORFs, and all other ORFs. Bars are ordered from ancient to recent, left to right. For visual purposes, only phylogenetic branches corresponding to named taxonomic groups are shown.

We compared the properties of high-confidence ancient ORFs, where all methodologies agreed that the node of origin is Anc5 (*n* = 442), with those of low-confidence (but potentially) ancient ORFs where only one or two methodologies predict an origin at Anc5 (*n* = 273). We found that high-confidence ancient ORFs are on average longer and have more similar initial alignments which also contain fewer gaps, than low-confidence ancient ones (Fig. 4a). This supports the status of the high-confidence ones as more robustly ancient since it is suggestive of conservation of a true protein-coding ORF. Low-confidence ancient ORFs have also significantly lower maximum RFC scores in their Anc5-reconstructed ancestor (Fig. 4a). In other words, either their best-reconstructed ORFs are much shorter than the extant *S. cerevisiae* one, or they are long but do not align well on the same frame as the extant *S. cerevisiae* ORF. Note here that 70 of the high-confidence ancient ORFs have relatively low best RFC score (<0.5), yet thanks to our empirical *P*-value we can confidently classify them as ancient. Finally, the posterior probabilities of the Anc5 reconstruction (as predicted by FastML_marginal) are lower both for indels and for individual positions (Fig. 4a). Thus, low-confidence ancient ORFs should be viewed as much harder cases than high-confidence ancient ones, with borderline predictions of their phylogenetic origins.

While informative regarding the limitations of ASR, these differences between high-confidence and low-confidence ancient ORFs do not allow to propose a most likely evolutionary scenario for the origin of the latter. Indeed, we would expect to see the same set of differences if low-confidence ancient ORFs were fast-evolving ancient ORFs for which only some of the methods happened to successfully capture their ancient status, or if they were recently emerged ORFs from regions of the genome where the probability of forming an ORF was high, making detection of a long enough in-frame ORF in one of the reconstructions more likely.

A relatively independent approach to estimate the timing of origination of a gene is protein-level phylostratigraphy (Domazet-Lošo et al. 2007). We analyzed protein-level sequence similarity searches against all available fungal proteomes (see Materials and Methods) and obtained, for each *S. cerevisiae* ORF, a phylostratigraphic node of origin (most recent common ancestor of species with match in the fungal tree). In Fig. 4b, we show the distribution of phylostratigraphic origins in the high-confidence and low-confidence ancient ORFs as well as for the other ORFs analyzed.

Most of the low-confidence ancient ORFs were *S. cerevisiae*-specific according to phylostratigraphy (64%). This percentage was significantly lower for high-confidence ancient ones (45.7%, *χ*^2^ test *P* = 0.011) yet still surprisingly high. Thus, for a significant percentage of ORFs, our ASR-based analyses point to a phylogenetic origin that is strikingly older than the one recovered by phylostratigraphy. This suggests that the origins established using our approach are conservative and best viewed as upper bounds. One possible explanation for this is that some of these ORFs might not be protein-coding at all (and hence correctly not annotated as such in other species) but could instead happen to overlap conserved noncoding elements. Another explanation is that their homologs have erroneously not been annotated in other species.

When looking at ORFs with an ancient phylostratigraphic origin, we find a much higher percentage among the high-confidence ancient ones (26% at Fungi) compared with the low-confidence ones (9.2%, *χ*^2^ test *P* = 7.7 ∗ 10^−6^). These trends suggest that the two groups, defined on the basis of ASR-predicted origin uncertainty, are qualitatively different, if only moderately. The origins of most of the low-confidence group as well as a significant percentage of the high-confidence one remain equivocal, because these results are what we would expect if they were mostly ancient but fast evolving, perhaps also easily lost genes, but it is also what we would expect if most of them were of truly recent origin.

Out of the 37 high-confidence *S. cerevisiae*-specific genes based on ASR, only four are not *S. cerevisiae*-specific according to phylostratigraphy. The first (YGR219W) has matches in only three distant species out of the thousands of fungal species contained in the target database, which strongly suggests this it is a *S. cerevisiae*-specific HGT. The second (YOL013W-A) has matches in 13 species, including *Saccharomyces eubayanus* and *Saccharomyces uvarum* but not in other *Saccharomyces*. This is an interesting case, as this gene has a paralog on the same chromosome (YOR072W-B, which also only has homologs in six species) and both overlap proline tRNA genes. The *S. uvarum* protein match is within part of a much longer protein (N7582_004276; 349 aa compared with 63 aa) that is not located in the orthologous region of YOL013W-A (which is found in chromosome XV) but in chromosome XIII. It is thus not surprising that ASR does not capture the ancient status of this ORF, whose evolution seems to include duplication and pseudogenization. The third gene (YLR390W) has matches with 266 species including all Saccharomyces; thus, it is clearly a conserved gene. Inspection of the orthologous alignment revealed that there is a strongly conserved part of the alignment that starts downstream of the annotated start codon in *S. cerevisiae*, where there is a conserved start codon in all species. Thus in this case the inclusion of this additional sequence segment contributed to the erroneous result (interestingly, FastML marginal predicts the ORF as being present at the root, but these reconstructions were not taken into account). The final gene (YBL112C) has homologs in 139 species including *Saccharomyces*, but here again, this is a protein with paralogs, as it is contained within the telomeric helicase-encoding Y element, which is found in many *S. cerevisiae* chromosomes. Its identified homologs are not within its orthologous regions, and the alignment of its orthologous regions shows no signs of conservation. Overall then, out of these four cases, only one presents a true ASR false positive.

## Discussion

ASR is a promising approach allowing to peek into the evolutionary past of sequences and elucidate the process of de novo gene origination. It has the potential to provide important novel insights both into the frequency and the evolutionary forces that drive de novo gene emergence. Nonetheless, it has been demonstrated that ASR is sensitive to many factors, including the methodology of multiple sequence alignment and the phylogeny (Vialle et al. 2018; Holmes 2017). In addition, other biases might come into play when the ancestral sequences are examined for the presence of relevant ORFs in the context of de novo emergence. For these reasons, we performed a systematic examination and assessment of ASR for the study of de novo gene origination.

Overall, we find that ASR is well suited to be used as a tool for the inference of de novo origination and that the variability in the results from the different methodologies is limited. With the notable exception of the marginal reconstructions of FastML, the rest of the methodologies were for the most part in good agreement as per the node of origin of an ORF relative to random expectations. It is possible that, while in the context of ancestral protein sequence resurrection, slight variations might lead to changes in functionally critical amino acids (e.g. that could affect the active site of a resurrected enzyme), the same slight variations might not impact the inference of the timing of origination of an ORF in the context of the present study.

One limitation of our approach stems from the crude null model that we employed that relies on pseudorandomization of genomic loci. Real evolution of genomic ORFs almost certainly deviates from this simplistic model, including by being slower due to overlap with other functional genomic elements. Future work could focus on reconstructing the evolution of real noncoding genomic ORFs to provide a more accurate estimation of their stochastic disruption along the tree.

An important point is that while we had one part of a good positive control set in genes with widespread presence and protein-level conservation in other species, we lack an independently generated “gold standard” set of de novo genes to compare to, and, most importantly, we lack an appropriate negative control. A potential solution to the latter would be to establish a gold standard collection of pseudogenes or, if this is not available, generate them through evolutionary simulations. These would represent evolution in the opposite direction of de novo emergence (gene death vs. gene birth), and they would be valuable as a test to our ASR-based workflow. For what percentage of such pseudogenes would we be able to accurately reconstruct their ancestral protein-coding status and the timing of their pseudogenization? This could be the focus of future work. At the same time, such work could also address the generalizability of the present findings in other lineages, including ones experiencing slower or faster evolution.

We believe that it is always best to start from a conservative place. Our empirical *P*-value approach might in fact be *too* conservative in considering all in-frame ORFs longer than would be expected randomly as potential evidence for selection. This is inextricably linked to the poorly understood questions of how de novo gene emergence begins, at which point during de novo gene evolution a protein is first expressed, and at which point the incipient ORF is subjected to selection at the level of its size. If the initial evolutionary “version” of a de novo gene has on average the same length as any spurious small ORF on the genome, then our assumption and the empirical *P*-value approach would be valid. If a slightly longer than usual small ORF is mostly what de novo emergence starts from, then it would be strongly conservative, since such ORFs continuously appear and disappear in the genome throughout evolution. One can envision a future approach that incorporates this probability into the calculation of a *P*-value to make it more realistic. Furthermore, it is important to consider that the evolutionary timing of the initial formation of an ORF might not coincide with it, or its wider locus, becoming protein-coding, which at the very least requires transcription and translation. This means that our estimated origins might not always correspond to the true origins of the *S. cerevisiae* protein-coding genes in question. Nonetheless, the consistency of the RFC scores and *P*-values in ancestors following the estimated branches of origin suggests that this should have limited impact on our conclusions.

## Supplementary Material

evae151_Supplementary_Data

## Data Availability

The study uses publicly available data. Data source tables are included as Supplementary material. Additional data and scripts are available at https://github.com/Nikos22/.
