# Correction to: Ancestral Sequence Reconstruction as a Tool to Detect and Study De Novo Gene Emergence

**DOI:** 10.1093/gbe/evaf059

**Published:** 2025-04-03

This is a **correction** to:

Nikolaos Vakirlis, Omer Acar, Vijay Cherupally, Anne-Ruxandra Carvunis, Ancxestral Sequence Reconstruction as a Tool to Detect and Study De Novo Gene Emergence, *Genome Biology and Evolution*, Volume 16, Issue 8, August 2024, evae151, https://doi.org/10.1093/gbe/evae151

In the originally published version of this manuscript, corresponding author, Nikolaos Vakirlis’, email address was presented with the wrong domain. The correct email is:

vakirlis@fleming.gr

This error has been corrected.

