# Supplementary material for: Ancestral Sequence Reconstruction as a Tool to Detect and Study De Novo Gene Emergence
Source: Genome Biol Evol. 2024 Jul 15;16(8):evae151. doi: 10.1093/gbe/evae151 (PMC11299112; doi:10.1093/gbe/evae151)
Supplement: evae151_Supplementary_Data [file evae151_supplementary_data.zip › Supplementary_Material.pdf]

Supplementary Figures

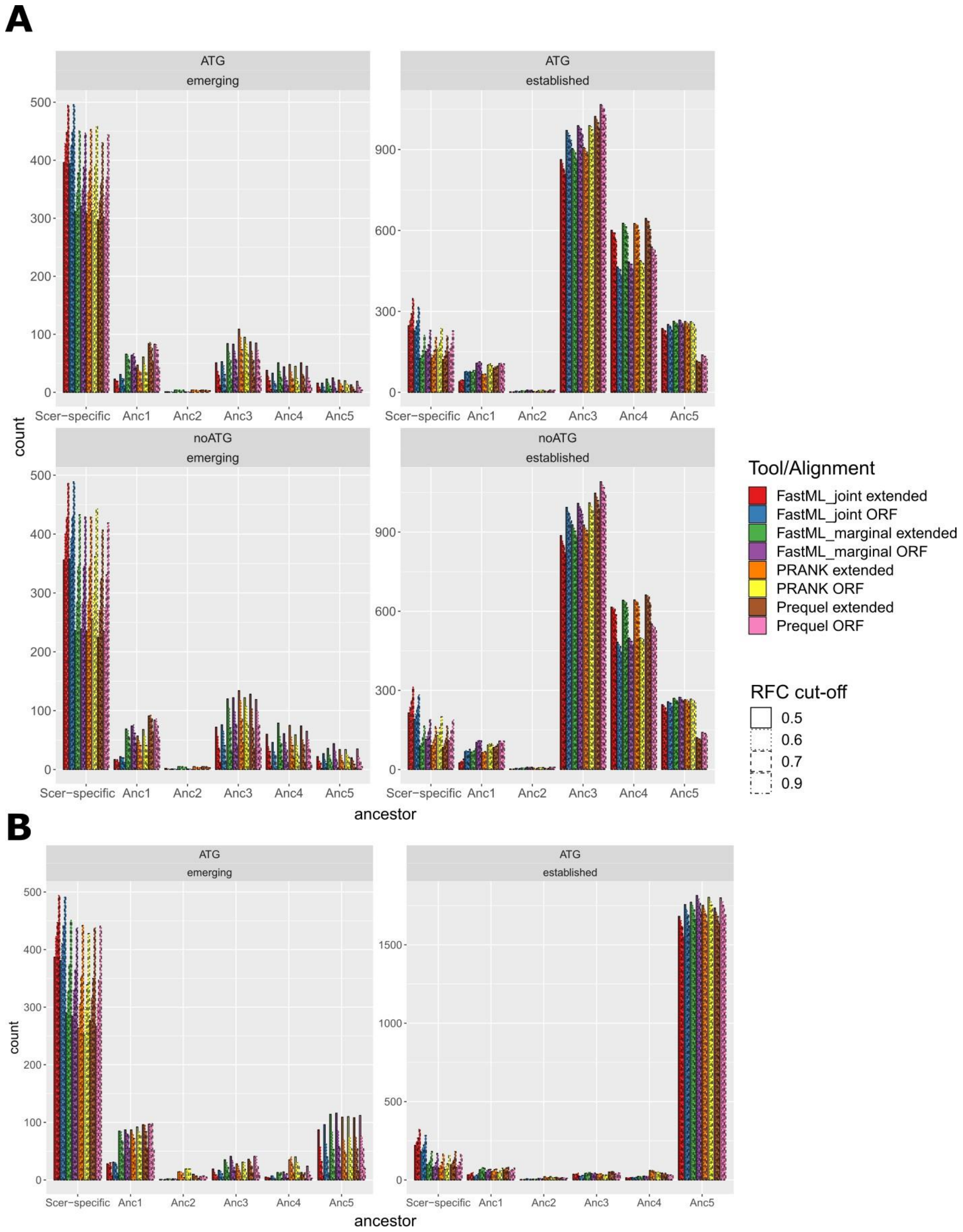

Supplementary Figure 1: **A**: Distributions of branch where the most ancient ancestral ORF has been identified using four different RFC cut-offs and eight different ASR methodologies (tools+alignment). Bars within each node (e.g. "Anc5") correspond, from left to right, to RFC cut-off of 0.5, 0.6, 0.7 and 0.9. Results for species topology using both "noATG" and "ATG" definition of ORFs are shown. **B:** Same as A but using the species topology and the "ATG" definition of ORFs.

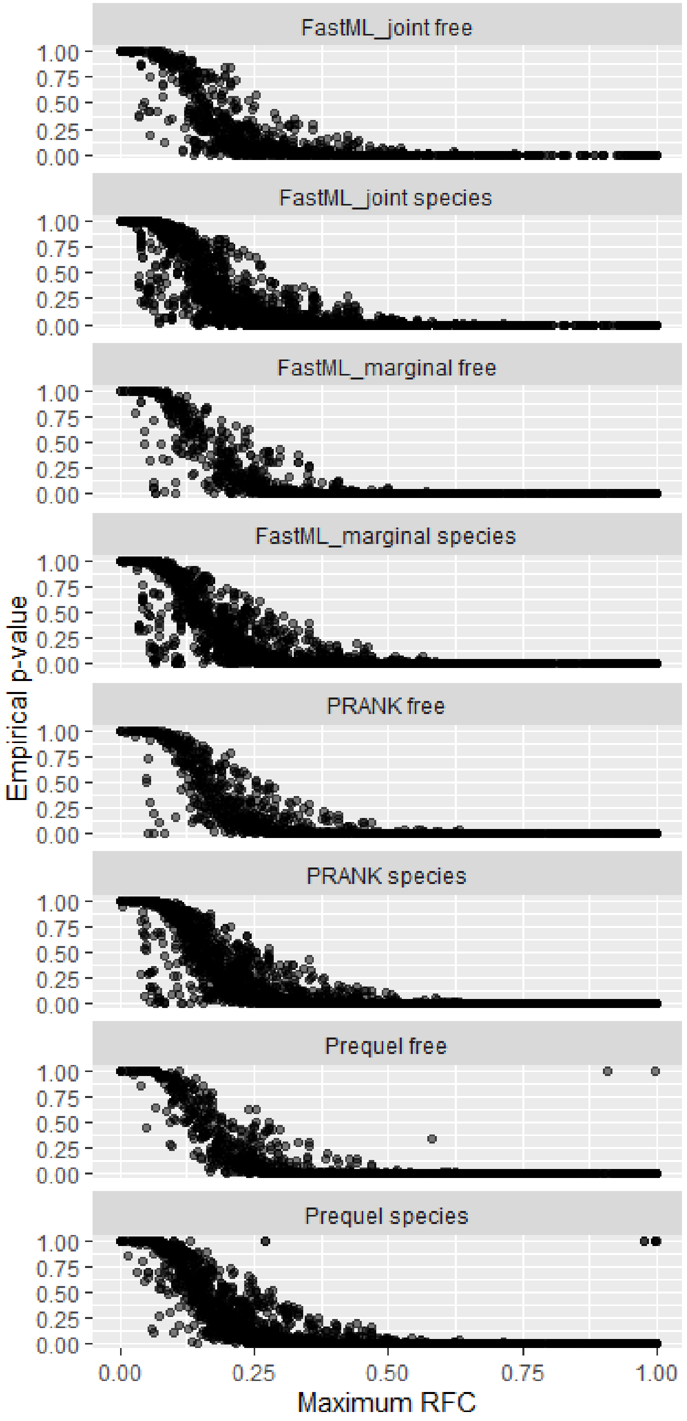

Supplementary Figure 2: Relationship of the maximum (best) RFC score in the Anc5 ancestor and the associated empirical P-value for different methodologies, using the ORF-only alignments and the "noATG" ORF definition.

Supplementary Tables

Supplementary Table 1. Gene names and associated data for 1,554 robustly ancient ORFs.

Supplementary Table 2. Best RFC and associated P-values for every ancestor, for the different methodological variations for 1,076 ORFs.
